# Combination of *teg1* and *our1* enhances total root length through preferential carbon allocation to lateral root growth in rice

**DOI:** 10.3389/fpls.2026.1914320

**Published:** 2026-07-23

**Authors:** Patrick Louie G. Lipio, Yihao Dong, Cornelius Mbathi Wainaina, Ryohei Sugita, Via Ann C. Marcelo, Mana Kano-Nakata, Nonawin Lucob-Agustin, Roel R. Suralta, Jonathan M. Niones, Yoshiaki Inukai

**Affiliations:** 1 Graduate School of Bioagricultural Sciences, Nagoya University, Nagoya, Aichi, Japan; 2 International Center for Research and Education in Agriculture, Nagoya University, Nagoya, Aichi, Japan; 3 Department of Horticulture and Food Security, Jomo Kenyatta University of Agriculture and Technology, Nairobi, Kenya; 4 Radioscope Research Center, Nagoya University, Nagoya, Aichi, Japan; 5 Philippine Rice Research Institute, Central Experiment Station, Science City of Munoz, Nueva Ecija, Philippines

**Keywords:** carbon distribution, lateral roots, mutant, rice, root system architecture

## Abstract

Root system development is essential for water and nutrient acquisition but requires substantial carbon investment. Optimizing root architecture while minimizing carbon costs is therefore an important target for crop improvement. Here, we characterized a novel rice (*Oryza sativa* L.) mutant, *truncated elongation growth 1* (*teg1*), which exhibits reduced seminal and crown root elongation but enhanced lateral root development. Histological analyses revealed that the shortened root phenotype was associated with reduced root apical meristem activity and decreased cell elongation. Rice possesses different lateral root types, with L-type lateral roots being thicker and capable of branching into smaller lateral roots, and S-type lateral roots being thinner and shorter than L-type lateral roots. Despite shorter seminal and crown roots, *teg1* developed a higher density of L-type lateral roots and longer L-type lateral roots than the wild type. To investigate the interaction between root architecture and carbon allocation, *teg1* was combined with the *outstanding rooting 1* (*our1*) mutation, which promotes seminal, crown and lateral root elongation. The *teg1 our1* double mutant exhibited increased L-type lateral root growth accompanied by reduced seminal and crown root growth while maintaining total root length. Carbon-14 tracer analysis revealed enhanced accumulation of newly assimilated carbon in elongating L-type lateral roots of the double mutant. Furthermore, the root-to-shoot dry weight ratio of the double mutant remained comparable to that of the wild type. These results suggest that *teg1* promotes compensatory lateral root development and alters carbon allocation within the root system, providing a potential genetic resource for improving root system architecture in rice.

## 1 Introduction

Rice (*Oryza sativa* L.) is a staple crop that provides a major source of calories for more than half of the world’s population, particularly in Asia. However, a substantial proportion of rice-growing areas are rainfed and frequently experience fluctuations in soil water availability, resulting in drought stress and unstable grain production (Dawe et al., 2010; Pandey and Bhandari, 2009). Improving root system architecture (RSA) has therefore become an important target for enhancing crop adaptation to water-limited environments (Farooq et al., 2009; Rogers and Benfey, 2015).

Root systems play a central role in water and nutrient acquisition and contribute significantly to plant performance under drought conditions. Previous studies have demonstrated that plants possessing larger or more effectively distributed root systems maintain higher biomass production and grain yield under water-deficit conditions (Lucob-Agustin et al., 2021; Panda et al., 2021; Serraj et al., 2011). In environments where soil physical constraints limit deep rooting, lateral root development becomes particularly important because it enables efficient exploration of upper soil layers and enhances water uptake under transient drought stress (Suralta et al., 2010; Lucob-Agustin et al., 2021).

The rice root system is composed of several morphologically and functionally distinct root types. The seminal root emerges during germination and supports early seedling establishment, whereas crown roots develop from stem nodes and constitute the major framework of the mature root system. Lateral roots, which are classified into L-type and S-type lateral roots, arise from both seminal and crown roots and substantially increase the soil-exploration capacity of the root system (Watanabe et al., 2020; Yamauchi et al., 1996). Among these, L-type lateral roots are particularly important because they possess the capacity for extensive elongation and further branching, thereby contributing substantially to total root length.

Genetic modification of RSA has emerged as a promising strategy for improving crop performance under environmental stress. Several rice mutants affecting root angle, root elongation, and lateral root formation have been reported to enhance adaptation to abiotic stresses (Hanazawa et al., 2013; Hasegawa et al., 2021, 2022; Lucob-Agustin et al., 2020a; Xiong et al., 2025; Zhu et al., 2012). One such mutant is *outstanding rooting 1* (*our1*), which exhibits enhanced root development through increased lateral root formation (Hasegawa et al., 2021, 2022; Dong et al., 2025).

Although larger root systems generally improve resource acquisition, their development requires considerable carbon investment. Carbon assimilated through photosynthesis must be partitioned between shoots and roots, and excessive allocation to root growth may compromise aboveground development and yield formation (Nada and Abogadallah, 2018; Wilson, 1988; Yang et al., 2012). Consequently, improving root architecture without increasing carbon costs represents a major challenge for crop breeding. Understanding how carbon allocation influences the development of different root types may therefore provide new opportunities for optimizing RSA.

In the present study, we identified a novel rice mutant*, truncated elongation growth 1* (*teg1*), which exhibits reduced seminal and crown root elongation but enhanced development of L-type lateral roots. We further investigated the genetic interaction between *teg1* and *our1* and examined carbon allocation patterns using ^14^C tracer analysis. Our results demonstrate that *teg1* promotes compensatory lateral root development and, when combined with *our1*, generates a root system characterized by enhanced lateral root growth without increasing overall root biomass allocation. These findings provide new insights into the relationship between carbon partitioning and root architectural plasticity in rice.

## 2 Materials and methods

### 2.1 Plant materials and growth conditions

The *teg1* mutant was obtained by mutagenizing *Oryza sativa* cv. Nipponbare using *N*-methyl-*N*-nitrosourea (MNU), as previously described in (Inahashi et al., 2018). Rice seeds of the wild-type *Oryza sativa* cv. Nipponbare (WT), *teg1* mutant, *our1* mutant, and F_2_ plants derived from crosses between the mutants were pre-germinated in water mixed with fungicide (0.25% [w/v] benomyl benlate; Sumitomo Chemical Co.) and placed in a growth chamber (MLR-351; Sanyo) at 28 °C with continuous light for 24 hours, followed by rinsing and soaking for 48 hours in tap water filtered using a water purifier (MX600; TORAY Industries).

For the assessment of root characteristics of WT, *teg1, our1, teg1 our1* double mutant, germinated seeds of selected lines were grown in filtered tap water without any nutrient supplementation. *our1* mutant germinating seeds used for differential glucose assay were treated with various concentrations of glucose (Wako Chemicals, Tokyo, Japan) that include 0, 0.05 and 0.5 mM concentrations. Both setups were grown for 10 days in the growth chamber at 28°C, under conditions of continuous illumination at 350 μmol m^−2^ s^−1^ light intensity (PPFD). 10-day-old seedlings were harvested for biomass measurements and morphological analyses.

Germinated seedlings used for assessment of root length traits, WT, *teg1* mutant, *our1* mutant and *teg1 our1* double mutant, were grown in filtered tap water without any nutrient supplementation for 1 week followed by growth in 1/10 strength nutrient solution for two weeks (Colmer, 2003). These plants were grown under the same temperature and illumination conditions as described above.

Evaluating WT and *teg1* mutant at higher growth stages, seedlings were transferred to seedling plates containing soil premixed with fertilizer. Seedling plates were grown in a screenhouse for 52 days (maximum tillering stage of the plant) before being transferred to 5L plastic containers filled with soil. Plants were then left to grow in the screenhouse until panicle initiation. At this stage, plant height and panicle number were measured.

### 2.2 Morphological characterization

Initial root and shoot traits of WT and *teg1* mutant, including the length of the longest crown root, seminal root length, and plant height, were measured with a ruler, crown root number was counted manually. The number of different root types and the lengths of the longest L-type and S-type lateral root were measured using open-source image analysis software, ImageJ (Schneider et al., 2012) from root images captured with a digital camera (D90; Nikon, Japan). Global calibration was performed by measuring 1cm pixel distance of a picture of a ruler, included for scale.

For biomass measurements, shoots and roots of 10-day-old WT, teg1, our1, and teg1 our1 seedlings were separated. Ten seedlings were pooled as one biological replicate, and three independent biological replicates were analyzed for each genotype. Samples were dried at 70°C until a constant mass was reached and weighed using an analytical balance. The root-to-shoot dry weight ratio was calculated as the root dry weight divided by the shoot dry weight.

For total root analysis of 3-week-old plants and 10-day-old glucose-treated *our1* mutants, root samples were scanned at 720 dpi using an EPSON 10000XL A3 size scanner (Seiko Epson Corporation, USA). Whole root samples were used for three-week old plants, while 10-day-old *our1* seedlings were sampled for a 20-cm basal segment of the seminal root. Scanned images were then analyzed for root lengths using WinRHIZO v.2007d (Regent Instruments, Quebec, Canada) at a pixel value threshold set at 175. Seminal and crown root lengths with a thick diameter were classified as d > 0.3mm, while S-type lateral root lengths were classified as d < 0.1mm. L-type lateral root lengths fell in between these two previous measurements at 0.1 mm ≤ d ≤ 0.3 mm.

### 2.3 Map-based cloning, plasmid constructs and plant transformation

To map the causative gene of *teg1* mutant, we performed a linkage analysis using F_2_ plants derived from crosses between *teg1* mutant and *Oryza sativa* cv. Kasalath. For complementation of *teg1* mutation, the WT coding sequence (CDS) of Nipponbare was amplified in the region extending from -3000bp to +2055bp (considering the *TEG1* translation site as +1) and cloned into the pGWB4 vector (Nakagawa et al., 2007) to generate a *pTEG1:TEG1* construct. To analysis the expression pattern of *TEG1* gene, the a 3 kb genomic fragment upstream of the *TEG1* translation start codon (ATG defined as +1) was PCR-amplified from wild-type ‘Nipponbare’ genomic DNA and cloned it into the pGWB1 vector (Nakagawa et al., 2007). The generated fusion constructs were introduced into the EHA105 strain of *Agrobacterium tumefaciens* via electroporation. Subsequently, the *pTEG1:TEG1* construct was transformed into a *teg1* mutant, and the *pTEG1*:*NLS*-3 × *Venus* construct was transformed into WT plants via *Agrobacterium*-mediated transformation, as described previously (Hiei et al., 1994; Ozawa, 2009). Transgenic plants were selected on MS medium containing 50mg L^-1^ hygromycin at 30°C.

### 2.4 Histological analysis

Cell division in the seminal root tip was analyzed using the Click-iT EdU Alexa Fluor Imaging Kit (Invitrogen/Molecular Probes). Roots of 4-day-old seedlings were incubated in filtered water containing 5 μM EdU for 30 min in 15 mL Falcon tubes, and samples were prepared as described previously (Lucob-Agustin et al., 2020a). Fluorescence was observed using a laser scanning microscope (FV3000; Olympus). For cell length measurement, seminal roots of WT and *teg1* seedlings were fixed in FAA solution (37% formaldehyde:glacial acetic acid:100% ethanol:water = 1:1:12.6:5.4) for 24 hours, dehydrated through a graded ethanol series, cleared in salicylic acid, and observed using the same microscope. For Venus reporter observation, Venus fluorescence was excited at 488 nm and detected at 510–530 nm, whereas tissue autofluorescence was excited at 405 nm and detected at 450–470 nm.

### 2.5 ^14^Carbon treatment and imaging

This procedure follows the same methodology as previously stated in (Kaiho-Soma et al., 2025). 10-day-old seedlings were placed in acrylic cases (28 x 28 x 49 cm). A 5 mL vial was connected to a sealed acrylic case with a tube. In the vial is 5 MBq of ^14^C-sodium bicarbonate (PerkinElmer, NEC086H005MC) and lactic acid were mixed to generate ^14^CO_2_, which was introduced into the acrylic case. After 24 hours the ^14^CO_2_ was absorbed by the sodium hydroxide, and the acrylic case was opened to remove plants for sampling.

Roots of the plant were attached to a mounting board/covered in polyethylene wrap, and contacted with an imaging plate (Cytiva, BAS-IP MS 2040). After contact at 4°C for 30 minutes, images were acquired using an Imaging Analyzer (GE Healthcare, Amersham Typhoon).

### 2.6 Statistical analysis

Differences in morphological and anatomical characteristics between groups were compared using two-tailed Student’s *t*-test or one-way ANOVA followed by multiple-comparison Tukey’s test, as appropriate, using GraphPad Prism 11.

## 3 Results

### 3.1 The *teg1* mutant exhibits reduced seminal and crown root elongation but enhanced L-type lateral root development

**Figure 1:**
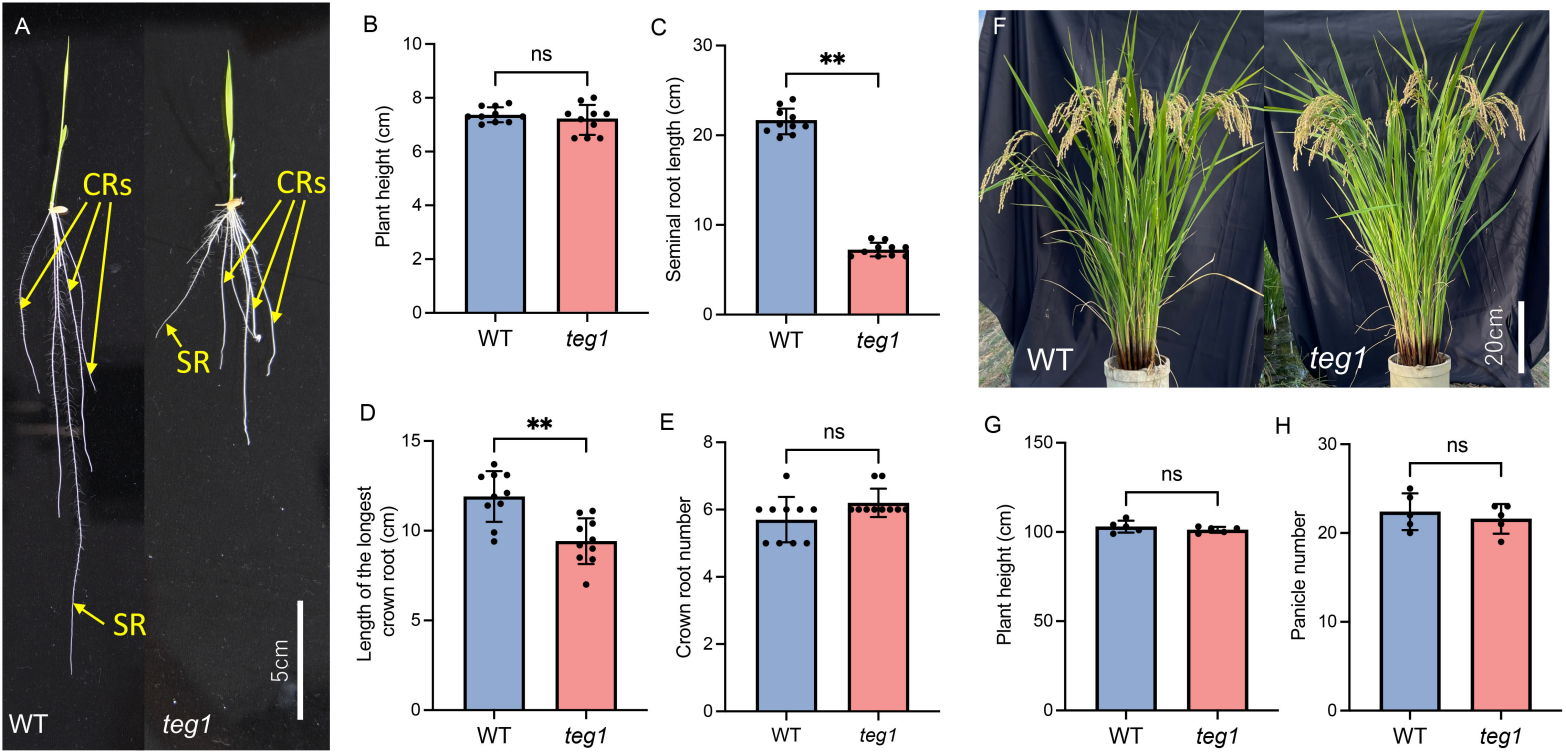
Phenotypic characterization of the *teg1* mutant. **(A)** Representative images of 10-day-old wild-type (WT) and *teg1* seedlings. SR, seminal root; CRs, crown roots. Scale bar = 5 cm. Seedling plant height **(B)**, seminal root length **(C)**, length of the longest crown root **(D)**, and crown root number **(E)**. **(F)** Representative images of mature WT and *teg1* plants. Scale bar = 20 cm. Plant height **(G)** and panicle number **(H)** of mature plants. Values are means ± SE (B–E, n = 10; G–H, n = 5). **P < 0.01 according to Student’s *t*-test; ns, not significant..

Initial phenotypic characterization revealed that the *teg1* mutant developed substantially shorter seminal and crown roots than the wild type (WT), whereas shoot growth was unaffected (**Figure 1A**). Quantitative measurements confirmed significant reduced seminal root length and length of the longest crown root in *teg1* (**Figures 1C, D**). In contrast, plant height and crown root number were comparable between WT and *teg1* seedlings (**Figures 1B, E**).

To determine whether the mutation had an effect on later developmental stages, mature plants were evaluated under soil-grown conditions. No significant differences were detected in plant height or panicle number between WT and *teg1* plants (**Figures 1F–H**), indicating that the mutation specifically affects root development without substantially altering aboveground growth.

**Figure 2:**
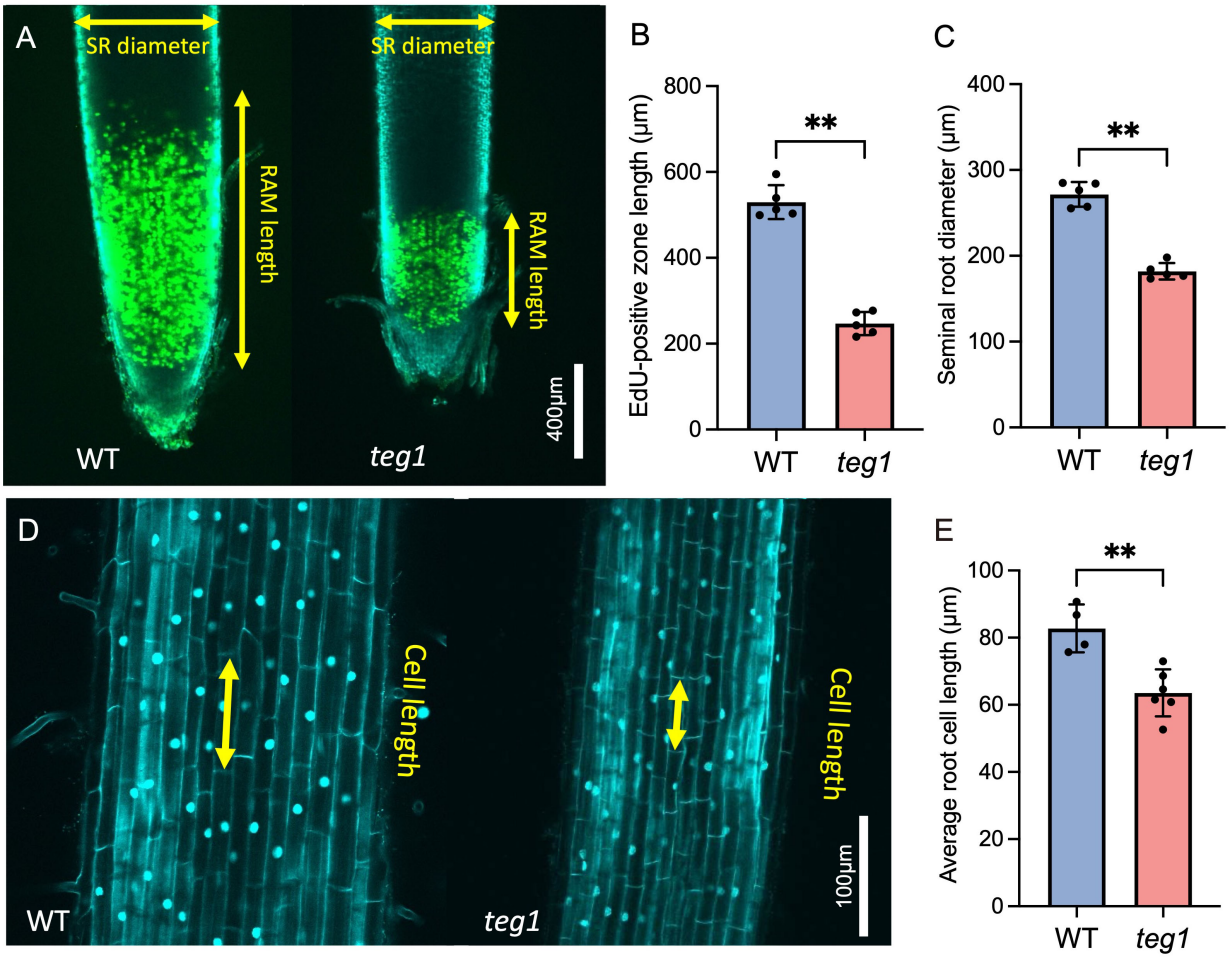
Reduced root apical meristem activity and cell elongation in the *teg1* mutant. **(A)** EdU staining of root apical meristems (RAMs) in 4-day-old WT and *teg1* seedlings. Green fluorescence indicates actively dividing cells. Scale bar = 400 μm. **(B)** RAM length and **(C)** root diameter. **(D)** Representative images of mature epidermal cells in seminal roots. **(E)** Epidermal cell length. Values are means ± SE (B–C, n = 5; E, WT n = 4, *teg1* n = 6). For cell-length measurements, five cells were measured per biological replicate. **P < 0.01 according to Student’s *t*-test. Yellow arrows indicate the regions used for measurement. Scale bar = 100 μm.

To investigate the cellular basis of the reduced root elongation phenotype, root apical meristem (RAM) activity was examined using EdU staining. The *teg1* mutant exhibited a markedly smaller region of actively dividing cells than WT (**Figure 2A**), resulting in a significant reduction in RAM length (**Figure 2B**). Seminal root diameter for the *teg1* mutant was also observed to be thinner than the WT (**Figure 2C**). Furthermore, mature epidermal cells of *teg1* were significantly shorter than those of WT (**Figures 2D, E**). These results indicate that both reduced cell proliferation and decreased cell elongation contribute to the shortened root phenotype of *teg1*.

**Figure 3:**
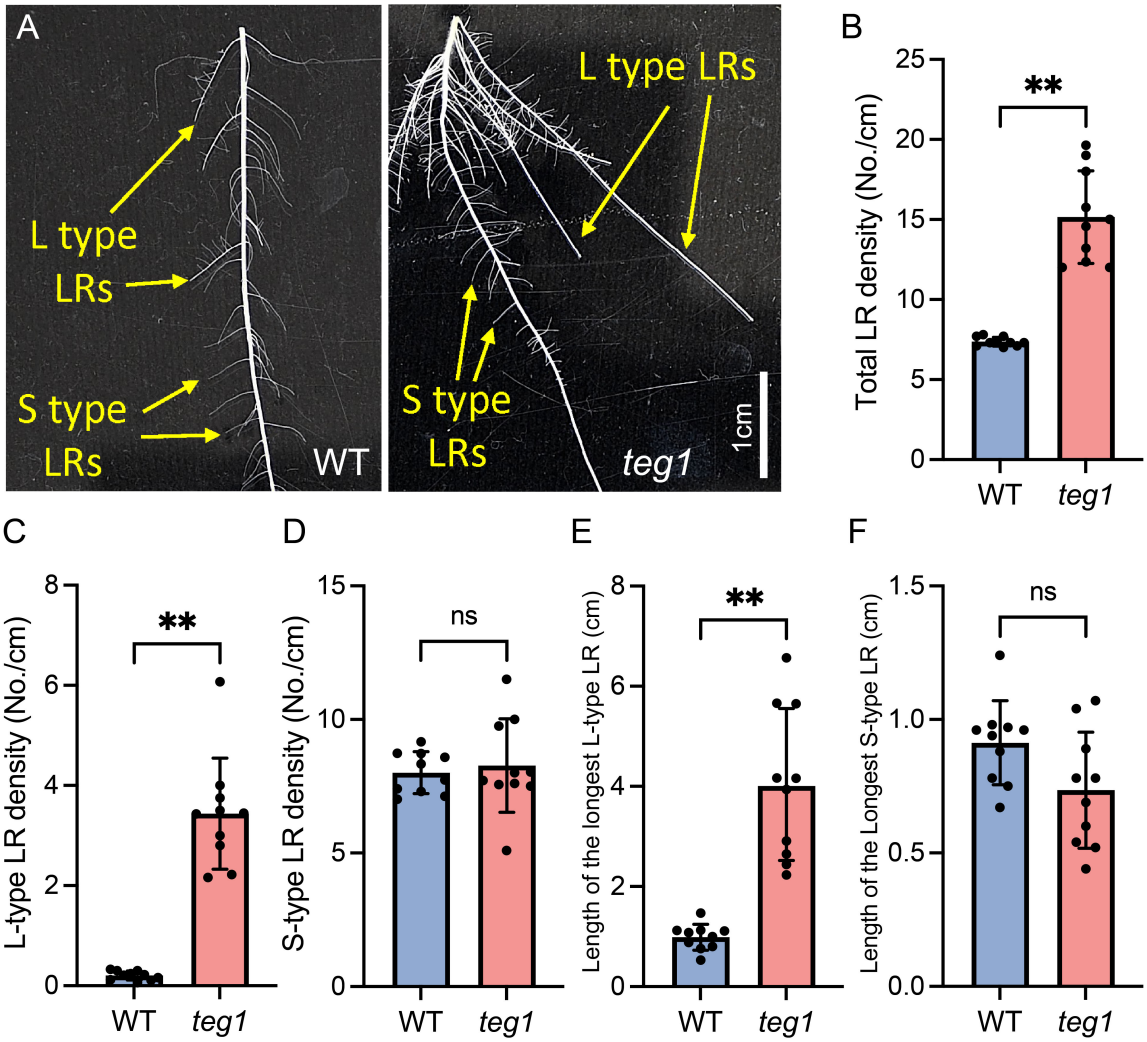
Enhanced L-type lateral root development in the *teg1* mutant. **(A)** Representative images of seminal roots from 10-day-old WT and *teg1* seedlings. Scale bar = 1 cm. Total lateral root density **(B)**, L-type lateral root density **(C)**, S-type lateral root density **(D)**, length of the longest L-type lateral root **(E)**, and length of the longest S-type lateral root **(F)**. LR, lateral root. Values are means ± SE (n = 10). **P < 0.01 according to Student’s *t*-test; ns, not significant.

Analysis of seminal root architecture revealed that *teg1* exhibited a significantly greater density of lateral roots than WT (**Figures 3A, B**). This increase was primarily attributable to L-type lateral roots, whose density was significantly higher in *teg1* (**Figure 3C**). In contrast, S-type lateral root density was unchanged (**Figure 3D**). Moreover, the length of the longest L-type lateral roots was significantly greater in *teg1* than in WT (**Figure 3E**), whereas the length of the longest S-type lateral root did not differ between genotypes (**Figure 3F**). These findings suggest that reduced seminal and crown root elongation in *teg1* is accompanied by enhanced L-type lateral root development.

### 3.2 *TEG1* encodes an ankyrin repeat-containing protein expressed in root meristematic tissues

**Figure 4:**
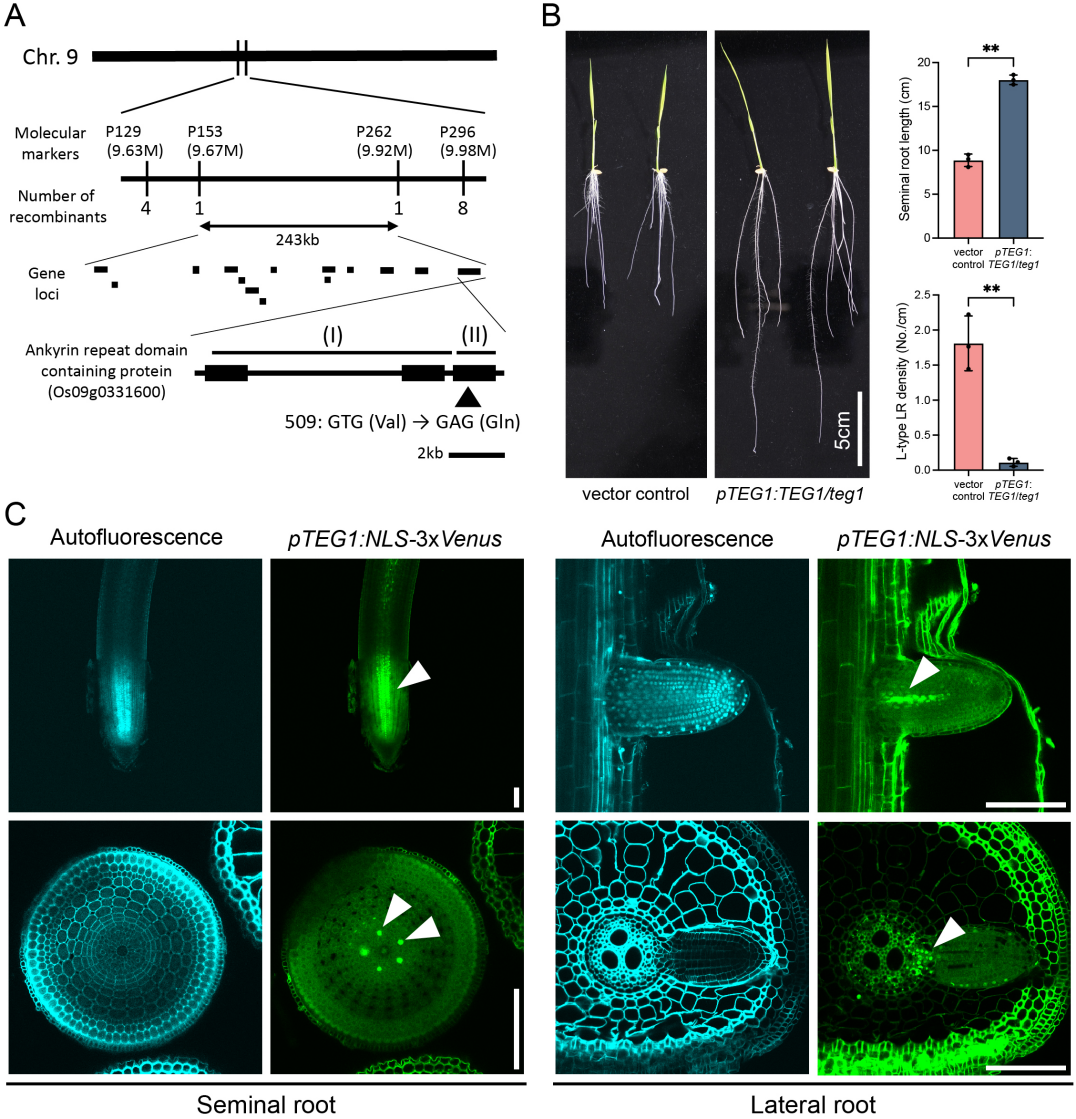
Gene isolation, complementation test and expression pattern of *TEG1* revealed by *pTEG1:NLS*-3×*Venus.* **(A)** High-resolution linkage map and genomic structure of *TEG1* (*Os09g0331600*) on chromosome 9. Black boxes and horizontal lines, in the gene model at the bottom, represent exons and introns, respectively. The arrowhead indicates an A-to-T nucleotide substitution in the third exon, resulting in a valine-to-glutamine amino acid substitution. Regions I and II indicate predicted ankyrin repeat-related domain and multiple transmembrane regions, respectively. **(B)** Complementation analysis of the *teg1* mutant. Representative images show the vector control in the *teg1* mutant background and a complemented line carrying the *pTEG1:TEG1* construct in the *teg1* mutant background. The graphs show quantification of seminal root length L-type lateral root density on the seminal root. **(C)** Expression pattern of *TEG1* revealed by the *pTEG1:NLS*-3×*Venus* reporter in longitudinal and transverse sections of seminal root apices and developing lateral root primordia. Venus fluorescence (green dots) denotes *pTEG1*-driven signal, and autofluorescence is visible in the background. White arrows indicate regions of *TEG1* expression. Scale bars = 100 μm.

Map-based cloning identified the causative gene of *teg1* mutant as *Os09g0331600*, an annotated gene located on chromosome 9, which we designated *TEG1* (**Figure 4A**). *TEG1* encodes an ankyrin repeat-containing protein with multiple predicted transmembrane regions. Sequence comparison between WT and *teg1* revealed a single A-to-T nucleotide substitution in the third exon, resulting in a valine-to-glutamine amino acid substitution within one of the predicted transmembrane regions (**Figure 4A**).

To confirm the identity of the causative gene, a complementation test was performed using the WT *TEG1* coding sequence. Introduction of the *pTEG1:TEG1* construct into the *teg1* mutant significantly increased seminal root length and reduced L-type lateral root density compared with the vector control (**Figure 4B**), suggesting that mutation of *TEG1* is responsible for the observed phenotype.

Expression analysis using a *pTEG1:NLS*-3 × *Venus* reporter revealed *TEG1* expression in the vascular tissues surrounding the root apical meristem of seminal roots and at the base of developing lateral root primordia (**Figure 4C**).

### 3.3 The *teg1* mutation alters carbon allocation patterns in the *our1* mutant background

**Figure 5:**
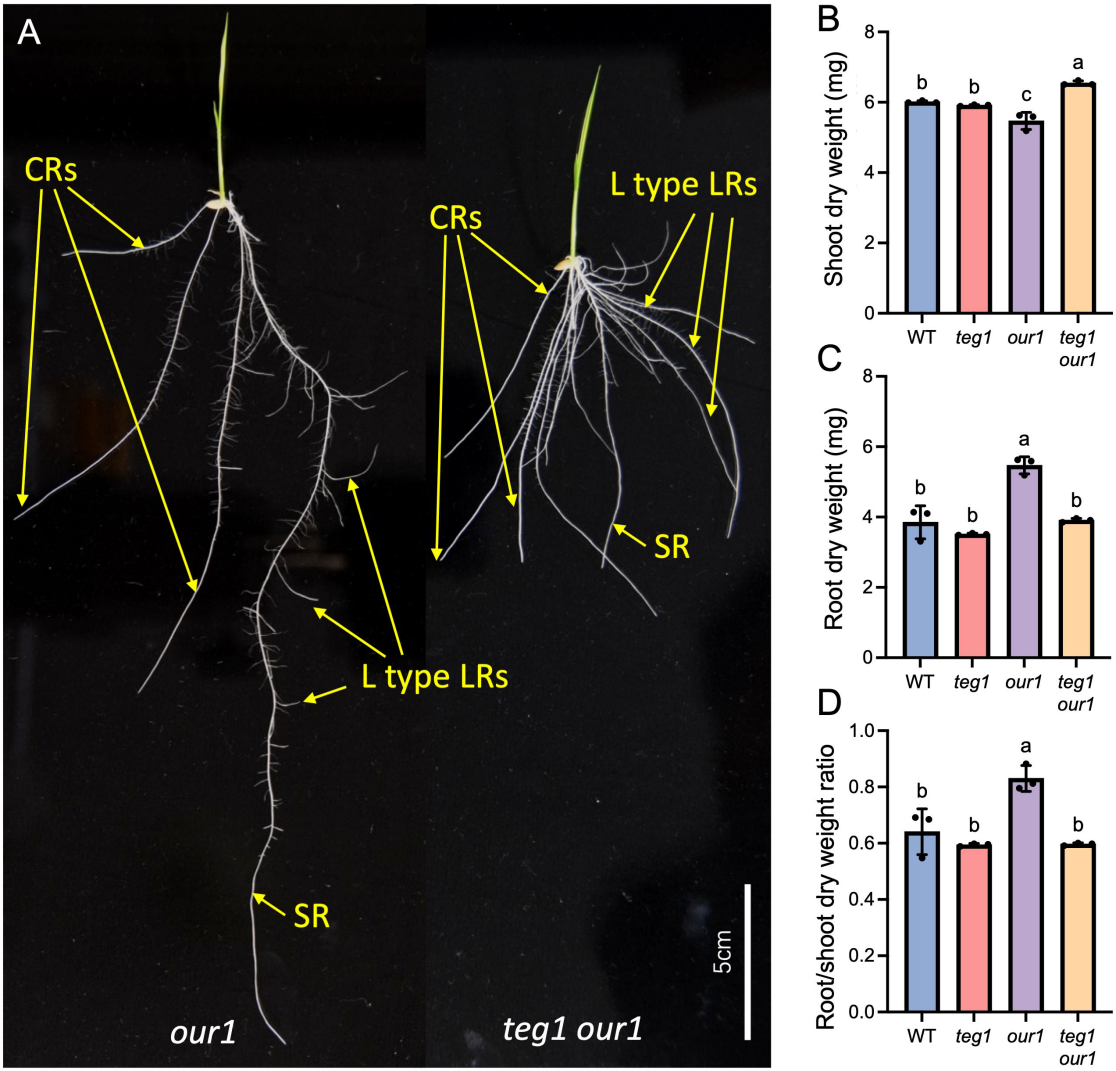
Root and shoot biomass allocation in WT, *teg1*, *our1*, and *teg1 our1* seedlings. **(A)** Representative images of 10-day-old *our1* and *teg1 our1* seedlings. SR, seminal root; CRs, crown roots; LRs, lateral roots. Scale bar = 5 cm. Shoot dry weight **(B)**, root dry weight **(C)**, and root-to-shoot dry weight ratio **(D)** of WT, *teg1*, *our1*, and *teg1 our1* seedlings. Values are means ± SE. Ten plants were pooled for each biological replicate (n = 3). Different letters indicate significant differences among genotypes according to one-way ANOVA followed by Tukey’s multiple-comparison test (P < 0.05).

To investigate the relationship between root architecture and carbon allocation, root and shoot dry weights were measured in WT*, teg1*, *our1*, and *teg1 our1* seedlings. Consistent with previous reports (Hasegawa et al., 2021), the *our1* mutant exhibited a longer seminal root and a greater number of L-type lateral roots than WT (**Figure 5A**). To evaluate the effect of the *teg1* mutation in the *our1* background, a *teg1 our1* double mutant was generated. Compared with the *our1* mutant, the double mutant exhibited shorter seminal and crown roots but enhanced development of L-type lateral roots (**Figure 5A**). Root and shoot dry weights, as well as the root-to-shoot dry weight ratio, were comparable between *teg1* and WT (**Figures 5B–D**). In contrast, the *our1* mutant exhibited greater root biomass and lower shoot biomass, resulting in a significantly increased root-to-shoot dry weight ratio (**Figures 5B–D**). Notably, the *teg1 our1* double mutant exhibited a root-to-shoot ratio comparable to that of WT despite its extensive L-type lateral root development (**Figures 5A, D**).

**Figure 6:**
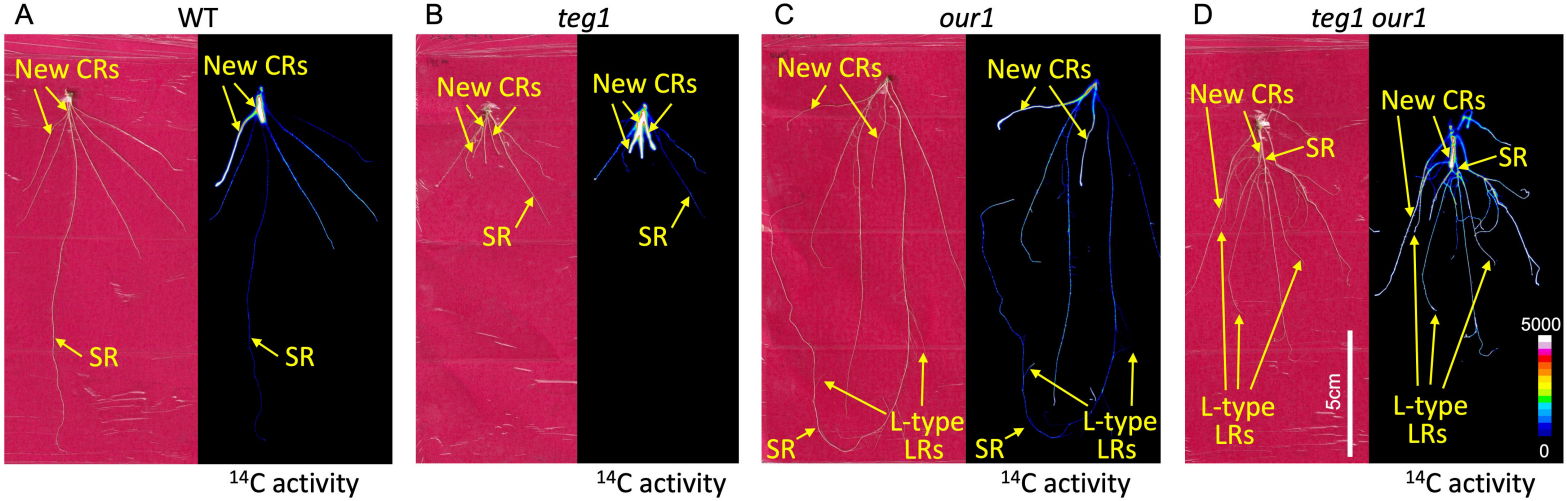
Distribution of newly assimilated carbon in rice root systems visualized by ^14^C tracer analysis. Representative root images (left, red background) and corresponding autoradiographs showing the distribution of newly assimilated ^14^C (right, black background), in 10-day-old **(A)** WT, **(B)** *teg1*, **(C)** *our1*, and **(D)** *teg1 our1* seedlings following ^14^CO_2_ feeding. The pseudocolor scale represents relative ^14^C signal intensity (0–5000 arbitrary units). Higher signal intensity indicates greater accumulation of newly assimilated ^14^C. SR, seminal root; CRs, crown roots; LRs, lateral roots. Scale bar = 5 cm.

Carbon allocation patterns were further examined using ^14^C tracer imaging. In WT, *teg1*, and *our1* plants, strong radioactive signals were predominantly detected in newly emerging crown roots (**Figures 6A–C**). In contrast, the *teg1 our1* double mutant exhibited substantial accumulation of newly assimilated carbon not only in newly formed crown roots but also throughout elongating L-type lateral roots (**Figure 6D**). These observations suggest that the combination of *teg1* and *our1* redirects carbon allocation toward L-type lateral root growth. If this hypothesis is correct, exogenous carbon application would be expected to further promote L-type lateral root elongation in the *our1* mutant.

**Figure 7:**
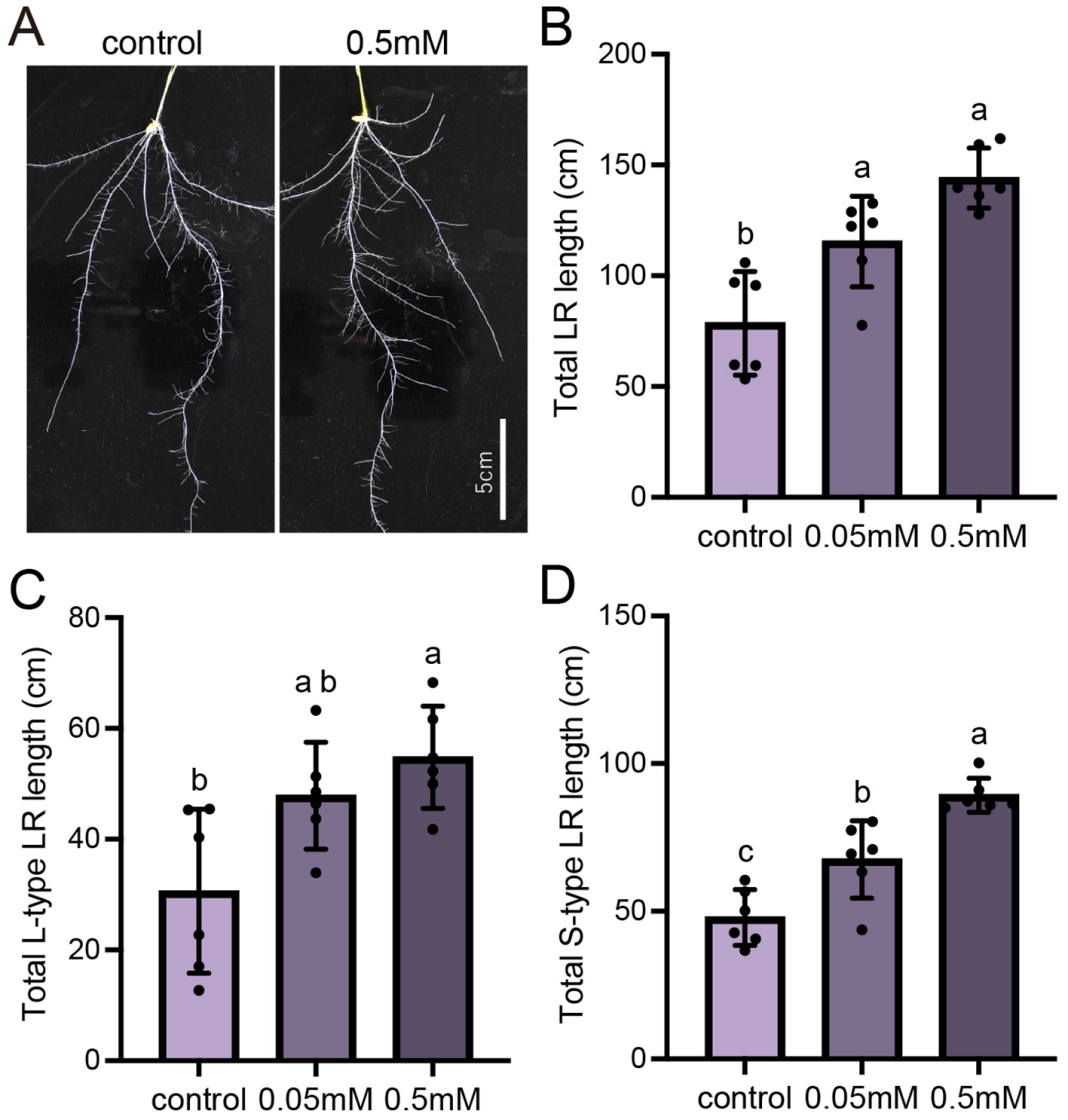
Effects of glucose treatment on lateral root development in the *our1* mutant. **(A)** Representative close-up images of lateral root growth in 10-day-old *our1* seedlings grown under control and 0.5 mM glucose conditions, respectively from left to right. Scale bar = 5cm. **(B-D)** Total lateral root length **(B)**, Total L-type lateral root length **(C)**, and Total S-type lateral root length **(D)** measured within a 20-cm seminal root segment. LR, lateral root. Values are means ± SE (n = 6). Different letters indicate significant differences among treatments according to one-way ANOVA followed by Tukey’s multiple-comparison test (P < 0.05).

Previous studies have shown that addition of exogenous carbon, glucose, resulted in the increase in lateral root length in rice (Lucob-Agustin et al., 2020b). To assess whether increased carbon availability promotes L-type lateral root elongation, *our1* seedlings were grown under different glucose concentrations. Increasing glucose concentration progressively enhanced total lateral root growth (**Figures 7A, B**). Both L-type and S-type lateral root lengths increased in response to glucose treatment (**Figures 7C, D**), indicating that carbon availability positively regulates lateral root elongation.

**Figure 8:**
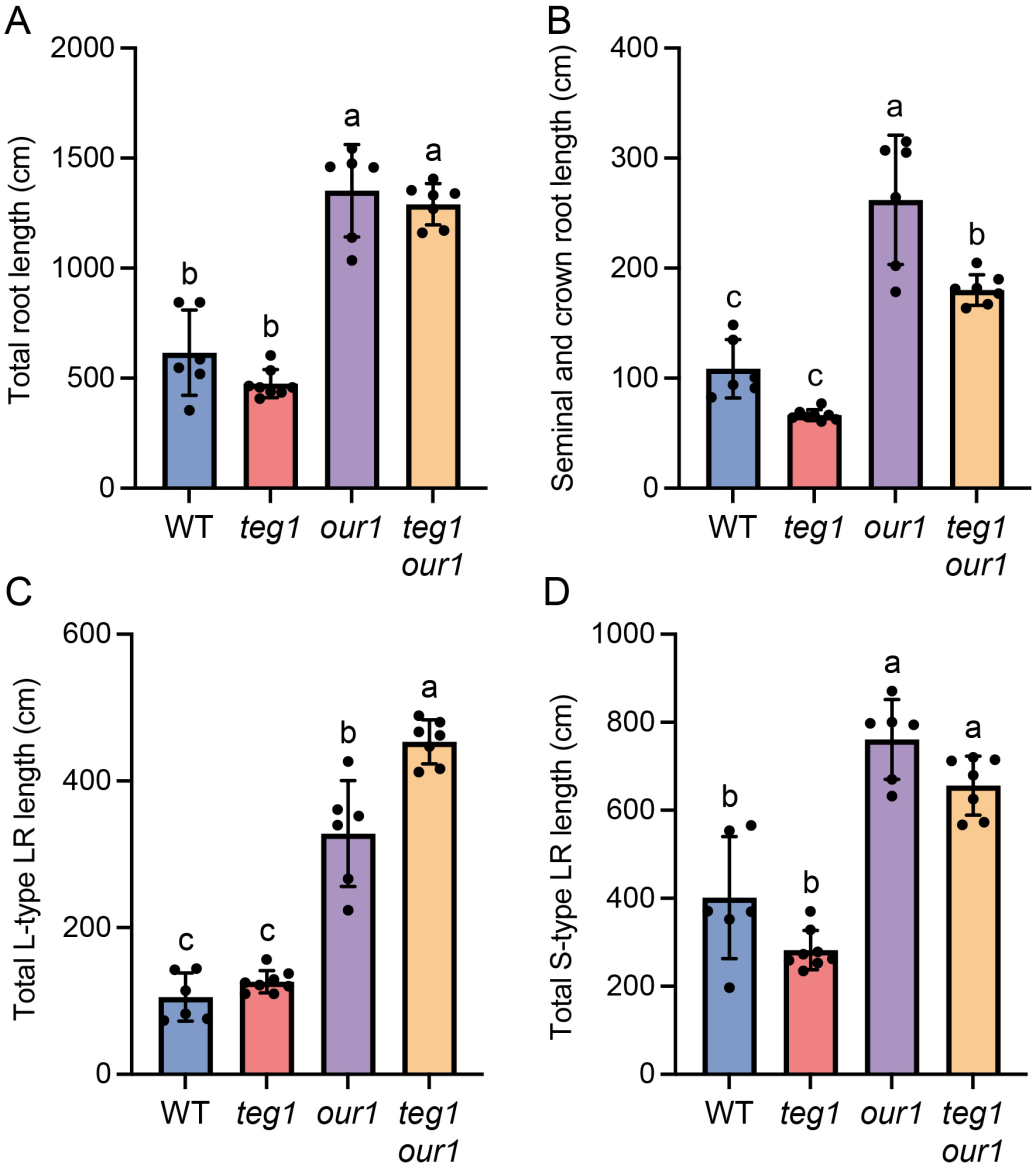
Root architectural characteristics of the *teg1 our1* double mutant in comparison to single mutants and wild type. Total root length **(A)**, seminal and crown root length **(B)**, total L-type lateral root length **(C)**, and total S-type lateral root length **(D)** in 3-week-old WT, *teg1*, *our1*, and *teg1 our1* plants. LR, lateral root. Values are means ± SE (WT n = 6, *teg1* n = 8, *our1* n = 6, *teg1 our1* n = 7). Different letters indicate significant differences among genotypes according to one-way ANOVA followed by Tukey’s multiple-comparison test (P < 0.05).

Root architectural traits were further evaluated in three-week-old plants. Total root length did not differ significantly between WT and *teg1* or between *our1* and *teg1 our1* (**Figure 8A**). However, partitioning total root length into individual root classes revealed marked differences in root system composition. Compared with the *our1* mutant, the *teg1 our1* double mutant exhibited reduced seminal and crown root length but significantly greater L-type lateral root length, whereas S-type lateral root length remained unchanged (**Figures 8B–D**). These results indicate that the *teg1* mutation promotes a redistribution of root growth from seminal and crown roots to L-type lateral roots while maintaining overall root system size.

## 4 Discussion

The present study identified and characterized a novel rice root mutant, *teg1*, which exhibits reduced seminal and crown root elongation but enhanced development of L-type lateral roots. When combined with the *our1* mutation, *teg1* generated a double mutant that maintained total root length through enhanced L-type lateral root elongation despite reduced seminal and crown root growth. These findings suggest that *teg1 our1* alters root growth patterns and carbon partitioning within the root system, resulting in enhanced lateral root development without increased root biomass allocation.

### 4.1 *TEG1* regulates root elongation and promotes compensatory lateral root development

The shortened seminal root phenotype of *teg1* appears to result from both reduced meristematic activity and decreased cell elongation. Map-based cloning identified *TEG1*, which encodes an ankyrin repeat-containing protein. Ankyrin repeat proteins participate in diverse biological processes through protein–protein interactions and have been implicated in the regulation of growth and development in both plants and animals (Huang et al., 2009; Li et al., 2006). The causative mutation in *teg1* results in a single amino acid substitution within a predicted transmembrane region of the protein. Because transmembrane domains are important for proper membrane localization and protein function, this mutation may impair the normal activity of TEG1. Furthermore, the expression of *TEG1* in root meristematic tissues is consistent with a role in regulating root elongation.

Another notable feature of the *teg1* mutant is the enhanced development and elongation of L-type lateral roots despite the reduction in seminal and crown root length. Similar responses have been reported in rice following physical restriction or excision of root apices, where suppression of primary root growth stimulates lateral root development as a compensatory response (Sasaki et al., 1984; Montagu et al., 2001; Kawai et al., 2017). Such compensatory growth has been proposed to maintain overall root system size despite limitations in the elongation of seminal and crown root axes (Kawai et al., 2022b). Consistent with this concept, *teg1* maintained total root length despite reduced seminal and crown root elongation through increased density and elongation of L-type lateral roots.

An alternative, although not mutually exclusive, explanation is that *TEG1* may directly participate in the regulation of lateral root development. This possibility is supported by the expression of *TEG1* at the base of developing lateral root primordia, a region where *OsWOX10*, a key regulator of L-type lateral root formation, is also expressed (Kawai et al., 2022a, 2022). Thus, the enhanced development of L-type lateral roots in *teg1* may not solely represent a compensatory response to reduced seminal and crown root growth but could also reflect a direct effect of the mutation on lateral root developmental processes.

Interestingly, although *TEG1* was expressed in both root apical meristems and developing lateral roots, the mutation negatively affected seminal and crown root elongation while promoting L-type lateral root development. This contrasting response may reflect differences in developmental regulation among root classes or functional redundancy among genes controlling lateral root growth (Li et al., 2022). Further studies will be required to elucidate the molecular mechanisms underlying the root-type-specific functions of *TEG1*.

### 4.2 The combination of *teg1* and *our1* alters carbon partitioning toward lateral root growth

The ^14^C tracer experiment provides insight into how the *teg1* mutation influences carbon partitioning within the root system. In WT and both single mutants, newly assimilated carbon was predominantly localized in newly emerging crown roots. In contrast, the *teg1 our1* double mutant exhibited strong accumulation of carbon in elongating lateral roots. This finding suggests that carbon allocation patterns were altered in the double mutant, resulting in preferential investment in lateral root growth. Such redistribution may arise from reduced carbon demand associated with shortened seminal and crown roots, thereby increasing carbon availability for lateral root elongation. The glucose treatment experiment further supports this interpretation, as increasing carbon availability promoted lateral root elongation in the *our1* mutant. Together, these observations suggest that carbon availability is a key factor regulating lateral root growth and that the combination of the *teg1* and *our1* mutations modifies the allocation of carbon resources among root classes.

An important outcome of this study is that the *teg1 our1* double mutant achieved extensive lateral root development without increasing the root-to-shoot biomass ratio relative to the wild type. In contrast, the *our1* mutant exhibited a substantially greater investment in root biomass. These findings suggest that the root systems of *teg1 our1* plants are constructed more efficiently than those of *our1* plants. Because L-type lateral roots generally require less biomass investment than crown roots while contributing substantially to soil exploration, a shift from crown root growth to lateral root growth may represent an energetically favorable strategy for root system expansion. Similar trade-offs between root architecture and carbon investment have been proposed in studies of root economics and resource acquisition efficiency (Farrar and Jones, 2008; Nielsen et al., 1994; Wang and Ruan, 2016).

### 4.3 Implications for the development of resource-efficient root systems

From an agronomic perspective, the ability to increase effective root system size without increasing carbon investment could be advantageous under resource-limited environments. Lateral roots contribute substantially to soil exploration, nutrient acquisition, and water uptake, particularly under drought conditions and in soils where deep rooting is physically constrained (Lucob-Agustin et al., 2021; Suralta et al., 2010). Therefore, the root architectural characteristics observed in the *teg1 our1* mutant may provide a useful framework for developing rice varieties with improved resource-use efficiency. Future studies should evaluate the performance of the double mutant under drought and nutrient-limited conditions to determine whether the altered root architecture translates into enhanced field performance. In conclusion, our results demonstrate that *teg1* promotes compensatory lateral root development and alters carbon partitioning within the root system. When combined with *our1*, the mutation generates a root architecture characterized by enhanced lateral root growth without increased biomass investment. These findings provide new insights into the relationship between carbon allocation and root architectural plasticity and may contribute to the development of resource-efficient root systems in rice.

## Data Availability

The original contributions presented in the study are included in the article. Further inquiries can be directed to the corresponding author.
